# Treatment of brain metastases from gastrointestinal primaries: Comparing whole-brain radiotherapy and stereotactic radiosurgery in terms of survival

**DOI:** 10.14744/nci.2021.65725

**Published:** 2021-02-11

**Authors:** Selvi Dincer, Defne Gurbuz

**Affiliations:** 1. Department of Radiation Oncology, Prof. Dr. Cemil Tascioglu City Hospital, Istanbul, Turkey; 2. Department of Radiology, Prof. Dr. Cemil Tascioglu City Hospital, Istanbul, Turkey

**Keywords:** Brain metastases, cumulative intracranial tumor volume, gastrointestinal cancer, prognostic factors, stereotactic radiosurgery, whole-brain radiotherapy

## Abstract

**Objective:**

The objective of the study was to analyze the clinical features and prognostic factors for survival in patients with brain metastasis (BM) from gastrointestinal primaries treated with whole-brain radiotherapy (WBRT) or stereotactic radiosurgery (SRS).

**Methods:**

We retrospectively investigated patients with BMs resulting from gastrointestinal primaries who underwent WBRT or SRS. The effects of treatment modalities on overall survival (OS) were calculated by the Kaplan–Meier method.

**Results:**

WBRT and SRS were applied to 24 and 17 patients, respectively. In the WBRT group, radiotherapy was delivered at 20–30 Gy in 5–10 fractions (fx). In the SRS group, a median dose of 22 Gy (range: 18–27 Gy) was applied in 1–3 fx. At BM diagnosis, all patients had synchronous extracranial metastases which were mostly detected in the lung and liver. Median OS values were 9 months and 4 months in the SRS and WBRT groups, respectively (p=0.005). Karnofsky performance status (KPS) score (≥70 vs. <70), diagnosis-specific graded prognostic index, gastrointestinal (GI) graded prognostic index, cumulative intracranial tumor volume (CITV), controlled systemic disease, and treatment modality (WBRT vs. SBRT) were found to be related with OS.

**Conclusion:**

In patients with GI cancer-related BMs, SRS should be preferred in those with longer OS expectancy who have controlled extracranial disease, good KPS and CITV values of <10 cm^3^.

**A**lthough gastrointestinal (GI) system malignancies are frequently observed, the incidence of brain metastases (BMs) is 4–8% [1]. While the most BMs resulting from GI malignancies are associated with colorectal cancers (1–4%), they are also detected in esophageal cancer (1.4–1.8%), stomach cancer (0.16–0.69%), liver cancer (1.3–2.9%), pancreatic cancer (0.1–0.3%), and gallbladder cancer (<0.5%) [2, 3]. Survival in GIS cancers has improved due to developments in the systemic treatment of primary disease, but the widespread use of radiological imaging techniques has increased the number of cases diagnosed with BM from GI primaries [4, 5].

Since BM from GI primaries is a rare occurrence, there are limited data concerning the management of such patients, especially with respect to risk factors, treatment modality, and overall survival (OS), and therefore, currently, there are no guidelines applicable to these patients. Median OS after BM has been reported to be 5.3 months (range: 2–9.6 months) [6]. However, advances in treatment options, such as better definition of prognostic factors in GI tumors, advances in surgical treatment, new chemotherapeutic agents, whole-brain radiotherapy (WBRT), and particularly stereotactic radiosurgery (SRS), have allowed the administration of relatively effective supportive treatment in patients with BMs. The use of multimodal therapy has been shown to result in considerably prolonged OS (41.1 months) [7, 8]. Furthermore, it is important to note that the development of BMs from GI tumors usually occurs in the late stage of the disease; thus, prognosis is affected by various factors including the progression of the systemic disease and the presence of lung and liver metastases [9, 10].

Patients with BM from GI primaries are a heterogeneous group and their outcomes vary greatly based on prognostic factors; therefore, several prognostic scoring systems have been developed to clarify pretreatment status and support decision-making for appropriate selection of therapy in each individual patient. The most widely used grading systems in cases of BM are the recursive partitioning analysis (RPA) scored with: Karnofsky performance status (KPS), age, controlled primary tumor and extracranial metastases (for those with non-specific primary cancer), and the diagnosis-specific graded prognostic index (ds-GPA) which adds the number of BMs instead of primary tumor status [11, 12]. In the ds-GPA model, KPS was found to be the only significant prognostic factor for cases with GI primaries [12, 13]. To improve the prognostic assessment of GI-related BMs, the GI-GPA was formed with the following factors: KPS, age, extracranial metastases, and number of BMs [14, 15]. Despite the fact that researchers have sought progress concerning this topic, neither the ds-GPA nor the gastrointestinal graded prognostic index (GI-GPA) takes into consideration the cumulative intracranial tumor volume (CITV), defined as the sum of BM volumes at the time of radiotherapy. Recently published data suggest that CITV could be an important predictor of treatment response and OS [16–18]; however, none of the available guidelines consider CITV value in their assessment.

As such, the aim of this study was to analyze various factors, including CITV and other characteristics, for their possible relationship with OS in patients with GI tumor-related BMs treated with WBRT or SRS.

**Highlight key points**

• BMs from GI primaries are seen in the late stage of disease and considered to be radioresistant tumors.
• To improve oncologic outcomes in this group of patients, WBRT and SRS are applied (used alone or in combination) but optimal treatment method has not been yet determined.
• Uncontrolled systemic disease, gastric primaries and KPS ≤70 are realted with poor prognosis.
• In patients with poor prognostic factors and short survival expectancy, WBRT should be preferred.
• For patients with longer OS expectancy who have controlled extracranial disease, good KPS and CITV values of <10 cc, SRS should be applied.

## Materials and Methods

### Patient Characteristics

The study population was defined as patients who suffered from BM development resulting from primary GI malignancies such as those involving the esophagus, stomach, pancreas, gallbladder, colon, and rectal region. Data concerning patients diagnosed with GI-related BMs, from January 2011 to December 2019, were retrospectively reviewed and recorded from the medical registry system of our hospital. This study was approved by the Institutional Review Board (approval date: August 28, 2020, approval number: 357). Patients with confirmed pathological diagnosis of primary GI malignancies (based on biopsy or surgical specimens) were included in the study. Exclusion criteria were the patients with secondary primary tumor, under the age of 18, who had previously undergone brain radiotherapy for a reason other than BM and previous metastasectomy. The diagnosis of BM with or without pathological evidence was retrospectively reconfirmed through contrast-enhanced computed tomography (CT) and/or magnetic resonance imaging (MRI) by a radiologist.

### Data Collection

Clinical data of the patients, including demographic characteristics, date of diagnosis, tumor histology, localization of the primary tumor, KPS, ds-GPA, and the features of the BM, were recorded. Patient files were evaluated in detail to determine time from initial diagnosis to BM development, presence of primary neurological symptoms or edema or hemorrhage due to BM, and the localization, number, and CITV of BM lesions (the latter defined as the sum of BM volumes [cc] at the time of radiotherapy). In addition, patients’ extracranial metastases were also evaluated from patient files, including presence/absence, time from the first diagnosis to the development of extracranial metastasis, localization, and the treatment modality applied. The GI-GPA was determined in patients according to age at diagnosis, presence of extracranial disease, KPS, and the number of BMs [14].

### Assessment of Treatment Response and Follow-up

In patients who received radiotherapy for BM, contrast-enhanced cranial CT and/or MRI studies were performed at the 1^st^, 3^rd^, 6^th^, 9^th^, and 12^th^ months during the 1^st^ year of follow-up. We evaluated OS with respect to the date of first treatment for BM. The patients were followed up by telephone and e-mail, as well as routinely scheduled follow-up studies in the outpatient clinic.

### Treatment Modalities

In the presence of BM, the treatment options afforded to patients were SRS and WBRT. The decision was based on the general condition of the patients, presence of neurological symptoms, and the number and location of BMs.

The SRS procedures were applied using a Cyberknife system (Accuray Inc., Sunnyvale, CA, USA) that was equipped with a 6 MV linear accelerator mounted on a computer-controlled robotic arm. All patients were treated in the supine position with a fitted thermoplastic mask for immobilization during simulation and treatment. CT images with 1 mm slice thickness were fused with contrast-enhanced MRI, and the clinical target volume (CTV) was defined as the enhanced lesion observed by contrast-enhanced MRI. By adding a margin of 2–3 mm to the CTV, the planning target volume (PTV) was generated. The brainstem, spinal cord, eyes, lenses, optic nerves, and the optic chiasm were contoured as organs-at-risk. The MultiPlan inverse treatment-planning algorithm (Accuray Inc.) was used for the generation of treatment plans. The dose was prescribed to 90% of the PTV. Biologically effective dose was calculated assuming an alpha/beta value of 10.

The WBRT approach was primarily taken in patients with multiple BMs (more than 5 metastases). Patients were immobilized in a supine position with a thermoplastic mask. The brain was contoured as a CTV equal to the PTV. Optic nerves, brainstem, eyes, and lenses were contoured as organs-at-risk. Planning CT was mandatory with a slice thickness of ≤5 mm. WBRT was performed with 6 MV photons using volume modulated arc therapy with a Varian linear accelerator (Siemens, Germany). The daily prescription dose was 3 and 4 Gy with a total dose of 20–30 Gy.

### Statistical Analysis

All statistical analyses were performed using the Statistical Package for the Social Sciences (SPSS) version 22 software (SPSS Inc., Chicago, IL, USA). Categorical variables are expressed using numbers (n) and percentages, while continuous variables are represented by the median and minimum-maximum values. The compliance of the numerical values to the normal distribution was examined using histograms or analytic methods (Shapiro–Wilk test). Since quantitative variables did not display normal distribution, two independent groups were compared using the Mann–Whitney U-test. Chi-square test was used to compare proportions between groups. Survival rates were calculated by Kaplan–Meier analysis. Factors affecting survival were analyzed with the log-rank test. The statistical significance level (alpha error) was set at 5%; therefore, p<0.05 was defined to be statistically significant.

## Results

### Patient Characteristics

Between January 2011 and December 2019, a total of 41 patients with BM from GI malignancies, mostly originating from colorectal and gastric tumors, were identified. Twenty-two were male and 19 were female. At initial diagnosis of primary GI tumors, median age was 62 years (range: 30–78 years), and the time from initial diagnosis to BM development was 13 (0–60) months (Table 1). Of the patients, 75.6% had a KPS of >70, and the GI-GPA scores ranged from 0 (24.4%) to 3 (7.3%).

**Table 1.** Patients’ and BM characteristics

	Total (n=41)
Characteristics of patients	
Age	
<65	61
≥65	39
Sex (%)	
Male	53.7
Female	46.3
Primary tumor	
Colon	22
Rectum	19.5
Stomach	39
Esophagus	4.9
Pancreas	9.8
Bile duct	2.4
Rectosigmoid	2.4
Histology	
Adenocarcinoma	90.2
Squamous	4.9
Neuroendocrine	4.9
KPS*	60–90
KPS	
≤70	26.8
>70	73.2
ds-GPA score	
ds-GPA = 1	24.4
ds-GPA = 2	43.9
ds-GPA = 3	31.7
ds-GPA = 4	0
GI-GPA score	
GI-GPA = 0	24.4
GI-GPA = 1	39
GI-GPA = 2	29.3
GI-GPA = 3	7.3
RPA score	
2	73.2
3	26.8
Systemic disease status at diagnosis of BM	
Controlled	24.4
Uncontrolled	75.6
Extracranial metastasis	
Lung	48.8
Bone	26.8
Liver	46.3
Systemic lymph node	29.3
Skin	2.4
Adrenal gland	7.3
Interval from diagnosi to extracranial metastasis*	0–49
Characteristics of BM	
Interval from diagnosis to BM* (months)	0–60
Number of metastasis	
1	39
2	19.5
≥3	41.5
Location of BM	
Supratentorial	48.8
Infratentorial	29.3
Both	22
Volume of BM (cc)*	0.34–112
Volume of BM (cc)	
<10 cc	51.2
≥10 cc	48.8
Edema	75.6
Hemorrhage	39
Neurological symptoms	
Headache	48.7
Ataxia	7.3
Motor weakness	29.3
Nausea	53.6
Mental change	14.6
Seizure	7.3
Asymptomatic	2.4

*: Median (minimum–maximum); BM: Brain metastasis; ds-GPA: Diagnosis-specific graded prognostic assessment; GI-GPA: Gastrointestinal graded prognostic assessment; KPS: Karnofsky performance status.

In 21 patients, synchronous extracranial metastases were detected in the liver and lung with the diagnosis of primary tumor; whereas all patients were found to have extracranial metastases at the diagnosis of BM. Extracranial metastases were most commonly detected in the lung (48.8%) and liver (46.3%). Imaging studies showed that 16 patients (39%) had solitary BMs, while 17 (41.3%) had three or more BMs. In SRS group, 10, 2, and 4 patients had 1, 2, and 3 metastases, respectively, while only one patient had four metastases. Overall, median CITV was 9.8 (0.34–112) cc. Twenty (48.8%) of the patients had a CITV value exceeding 10 cc.

### Treatment

WBRT was applied to 24 patients (58.5%), with a total dose of 30 Gy/10 fractions (fx) in 17 patients and 20 Gy/5 fx in seven patients. Seventeen patients underwent SRS with the margin dose of 18–27 Gy (median 22 Gy) in 1–3 fx (Table 2). The comparison of the patients treated with SRS and WBRT is summarized in Table 3. The patients whom selected for SRS treatment had better KPS, ds-GPA score, and GI-GPA score than the WBRT group. Furthermore, interval from diagnosis to BM was longer than those treated with WBRT (median 20 months vs. 9 months, respectively, p=0.008). However, CITV was lesser in the patients treated with SRS <10 cc had better OS in the SRS group of patients compared with the WBRT group.

**Table 2.** Treatment characteristics

Characteristic	%
Treatment modality	
WBRT	58.5
SRS	41.5
WBRT dose	
30 Gy/10 fx	41.5
20 Gy/5 fx	17
Dose of SRS*	18–27 Gy/(1–3) fx
GTV* (cc)	0.46–19.9

*: BM: Brain metastasis; GTV: Gross tumor volume; SRS: Stereotactic radiosurgery; WBRT: Whole-brain radiotherapy.

**Table 3.** Comparison of WBRT and SBRT

	WBRT (n=24)	SRS (n=17)	p
WBRT	SRS	p	(n=24)	(n=17)
Characteristics of patients	
Age			
<65	66.7	52.9	0.375
≥65	33.3	47.1	
Sex			
Male	33.3	82.4	0.002
Female	66.7	17.6	
Primary tumor			
Colon	8.3	41.2	0.027
Rectum	16.7	23.5	
Stomach	50	23.5	
Esophagus	4.2	5.9	
Pancreas	16.7	0	
Bile duct	0	5.9	
Rectosigmoid	4.2	0	
Histology			
Adenocarcinoma	87.5	94.1	
Squamous	4.2	5.9	
Neuroendocrine	8.3	0	
KPS*			0.003
KPS			
≤70	41.7	5.9	0.014
>70	58.3	94.1	
ds-GPA score			0.038
ds-GPA=1	37.5	5.9	
ds-GPA=2	41.7	47.1	
ds-GPA=3	20.8	47.1	
ds-GPA=4	0	0	
GI-GPA score			0.035
GI-GPA=0	37.5	5.9	
GI-GPA=1	41.7	35.3	
GI-GPA=2	16.7	47.1	
GI-GPA=3	4.2	11.8	
Systemic disease status at diagnosis of BM			
Controlled	20.8	29.4	0.714
Uncontrolled	79.2	70.6	
Extracranial metastasis			
Lung	37.5	68.7	0.053
Bone	25	35.7	0.712
Liver	54.2	46.2	0.642
Systemic lymph node	41.7	15.4	0.103
Skin	0	7.7	0.351
Adrenal gland	12.5	0	0.538
Interval from diagnosis to extracranial metastasis*	0 (r: 0–36)	10 (r: 0–49)	0.017
Median OS (months)	4	9	0.005
Characteristics of BM			
Interval from diagnosis to BM* (months)	9 (0–40)	20 (0–60)	0.008
Number of metastasis			
1	25	58.8	0.102
2	25	11.8	
≥3	50	29.4	
Location of BM			
Supratentorial	45.8	52.9	0.497
Infratentorial	25	35.3	
Both	29.2	11.8	
Volume of BM (cc)*	45.9 (r: 0.34–112)	6.4 (r: 0.46–19.9)	0.024
Volume of BM (cc)			
<10 cc	37.5	70.6	0.037
≥10 cc	62.5	29.4	
Edema	75	76.5	1
Hemorrhage	37.5	41.2	0.812

*: Median (minimum–maximum); BM: Brain metastasis; ds-GPA: Diagnosis-specific graded prognostic assessment; GI-GPA: Gastrointestinal graded prognostic assessment; KPS: Karnofsky performance status; OS: Overall survival; SRS: Stereotactic radiosurgery; WBRT: Whole-brain radiotherapy; r: Range.

Primary disease was under control in only 10 (24.4%) patients at the diagnosis of BM. All patients with extracranial metastases had previously undergone at least second-line chemotherapy.

### Factors Related with OS

**Figure 1.**
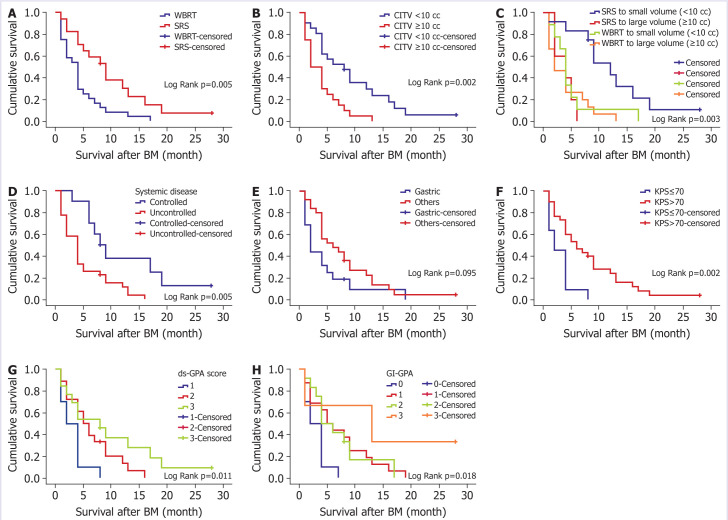
Prognostic factors related with overall survival. **(A)** Whole-brain radiotherapy (WBRT) versus stereotactic radiosurgery (SRS). **(B)** Cumulative intracranial tumor volume (CITV) <10 cm^3^ versus CITV ≥10 cm^3^. **(C)** SRS versus WBRT for small and large volume. **(D)** Controlled versus uncontrolled systemic disease. **(E)** Primary tumor originated from gastric cancer versus others. **(F)** Karnofsky performance status (KPS) ≤70 versus KPS >70. **(G)** Diagnosis-specific graded prognostic index (ds-GPA)=1 versus ds-GPA=2 versus ds-GPA=3. **(H)** Gastrointestinal graded prognostic assessment (GI-GPA)=0 versus GI-GPA=1 versus GI-GPA=2 versus GI-GPA=3.

Overall, the patients in whom BMs had developed from GI malignancy were followed up for a median period of 4 months (0–48 months) after WBRT and SRS. The median OS was 4 months. The survival time of patients treated with SRS was better than those treated with WBRT (median OS 9 months vs. 4 months, respectively, p=0.005) (Fig. 1A). Two-year survival rate was 7.6% in the SRS group, whereas none of the WBRT recipients survived for a duration of 2 years. Having a CITV <10 cc was associated with longer survival time compared those with a volume >10 cc. (median OS: 8 months vs. 2 months, respectively, p=0.002) (Fig. 1B). In addition, the patients who underwent SRS with a CITV of <10 cc had better survival compared to those who received WBRT with a CITV of ≥10 cc (p=0.003) (Fig. 1C). Patients with controlled disease had better OS compared to those with uncontrolled disease (median OS: 8 months vs. 4 months, respectively, p=0.005) (Fig. 1D).

While median OS was 2 months in patients with primary gastric cancer, it was 6 months in those with other GI cancers. Although there was no statistically significant difference, P-value approaching statistical significance was observed (p=0.095) (Fig. 1E). The median OS of patients with a KPS value exceeding 70 was 6 months, which was significantly longer than the median 2 months of OS in subjects with a KPS of ≤70 p=0.002) (Fig. 1F). There were significant differences in terms of survival according to different ds-GPA and GI-GPA prognostic scores (p=0.011 and p=0.018, respectively) (Fig. 1G, H). No statistical difference was found between the RPA prognostic groups (p=0.189).

## Discussion

The survival of patients with BMs from GI primary tumors is worse than those with BMs from breast cancer, lung cancer, melanoma, and renal cancer [19]. Development of BM in GI primaries is a rare event and occurs in the late stages of the disease. In addition, studies report that patients with BMs from GI primaries survive for a median of 5–6 months [20–22]. However, clear recommendations regarding which group of patients with GI tumor-related BMs could benefit from SRS (compared with conventional WBRT) have not been established. In this retrospective study, 41 patients with BMs from GI primaries who had undergone treatment with either WBRT or SRS were examined. Our analyses showed that KPS, ds-GPA, GI-GPA, CITV (<10 cc), presence of stable extracranial disease, and treatment modality (WBRT vs. SBRT) were associated with OS.

Patients with larger total CITV were less likely to achieve local control (LC) with SRS, and OS was likewise influenced by total volume in addition to systemic factors, such as the presence of extracranial disease, older age, and lower DS-GPA. Notably, the number of lesions was again not prognostic for either of these outcomes. The literature concerning the relationships between total tumor volume and response to treatment remains conflicted. Furthermore, the contribution of tumor volume on therapeutic success with SRS treatment is also a matter of debate. Thus, there are limited data to take into account when considering the utility of SRS according to CITV values.

The graded prognostic assessment has been validated for BMs resulting from solid tumors for its utility in aiding decision-making and classifying patients for appropriate treatment. The most common factors found to be associated with the worse OS were advanced age, low KPS, presence of extracranial systemic disease, and multiple BMs [6]. However, in the ds-GPA model, KPS was the only significant prognostic factor that was relevant for GI tumor-related BMs; however, new factors such as age, extracranial metastases, and number of BMs were added to the KPS factor, resulting in the establishment of the recently updated GI-GPA [12, 14]. Furthermore, some studies have evaluated KPS as a risk factor OS and have reported a survival benefit with higher KPS [23]. In the present study, OS results were found to correlate with ds-GPA and GI-GPA, and KPS ≥70 was found to be associated with prognosis; but advanced age and number of metastases were not determined to be prognostic factors with respect to OS.

In addition, uncontrolled extracranial disease affected OS in our study. In about 20–30% of patients with GI malignancy, the metastases are mostly found in the liver and lung, including those with synchronous and metachronous disease [24, 25]. Of note, the presence of uncontrolled extracranial disease has been suggested to be the most important predictive factor for distant brain failure which may act as an origin of distant seeding [26]. Thus, the presence of uncontrolled extracranial disease may reflect increased aggression with worse prognosis and could help clinicians decide on the approach to treatment in BMs. That is, whether SRS or WBRT would be applied without compromising the patients’ quality of life.

The current standard of care for patients with BMs consists of WBRT and/or SRS and/or surgery [27]. However, in BMs with a large mass effect, surgical resection rapidly diminishes neurological deficits, reduces the use of steroids, and provides good quality of life for patients [28]. Radiotherapy applied with WBRT and SRS (used alone or in combination) is an essential component of the treatment of BMs and has been associated with improving oncologic outcomes [29]. The benefits of the combined approach are mostly limited to a better rate of LC, but no survival benefit has been reported [27, 30]. Prior studies have suggested that, while selecting the optimal treatment method, the patient’s general condition, status of extracranial metastases, size/localization of metastatic brain tumors, and the number of brain lesions should be taken into consideration [13].

However, in a large prospective randomized study, comparing SRS alone versus SRS and WBRT in patients with 1–4 BMs found higher intracranial failure rates but no compromise on OS with SRS alone. Nonetheless, it was suggested that since WBRT may cause impairment in neurocognitive functions, it may be considered in patients with more than 1 metastatic lesion and a relatively shorter life expectancy [31]. Another prospective randomized study assigned patients with 1–3 BMs to WBRT and SRS or SRS alone, and defined the primary endpoint as neurocognitive functions (significant reduction compared to baseline). The researchers observed that withholding WBRT in favor of SRS alone was associated with improved neurocognition and increased survival, with the disadvantages of reduced local and distant control [32]. In a recent Phase III randomized controlled trial (JCOG0504), SRS was deemed to be non-inferior to WBRT since median OS was similar in both treatment arms. However, it was emphasized that more cognitive dysfunction was seen in the WBRT arm compared to the SRS arm. The authors concluded that SRS could be utilized for patients with fewer than 4 BMs [33]. It is well-established that making a decision regarding the radiotherapy modality for BM treatment is difficult for clinicians. In the present study, the patients treated with SRS survived longer than the patients treated with WBRT. This is undoubtedly associated with the fact that WBRT was mostly selected for patients with a life expectancy shorter than 6 months with multiple metastases, uncontrolled extracranial disease, and/or those with poor performance status; whereas, SRS was mostly was applied in patients with a longer survival expectancy who had KPS of >70, ≤3 metastases, and controlled systemic disease.

In addition, as GI tumors are particularly considered to be radioresistant, intense dose radiotherapy is preferred for higher LC without neurocognitive function impairment if SRS is to be applied for small volume and fewer metastases (e.g., 25 Gy) [34]. Notably, Trifiletti et al. [35] conducted an analysis of data from the largest series of patients diagnosed with GI cancer-related BMs in whom SRS was applied with a margin dose of ≥20 Gy. The authors reported a LC rate of 94.1% and a median survival time of 6.2 months. In the present study, the median margin dose was 22 Gy (range: 18–27 Gy) with the group of patients treated with SRS.

Recently, several investigators have suggested that CITV is a more relevant prognostic factor than the size or number of lesions, since greater tumor volume was found to correlate with worse OS [36–38]. Baschnagel et al. [39] concluded that patients with a total tumor volume >2 cm^3^ had worse OS, LC, and distant brain failure following SRS. Similarly, Hamel-Perreault et al. [16] found that, rather than the total number of BMs, the presence of CITV <6 cm^3^ and RPA Class I was independent prognostic factors associated with better OS. In the study by Routman et al. [18], the strongest prognostic factors for patients undergoing SRS were the tumor volume >10 cc and KPS, rather than the number of BMs. In a multi-institutional prospective study (JLGK0901), SRS was applied for patients with 1–10 BMs and a solitary tumor. The analyses demonstrated that female sex, age younger than 65 years, KPS of ≥80, stable extracranial disease, and lack of neurological symptoms significantly favored longer survival in multivariable analysis; however, having a CITV of <1.9 mL was found to be significantly favorable in only univariate analysis [23]. In a recently published trial evaluating patients with BMs from GI primaries, both CITV >12 cm^3^ and KPS were found to be independently associated with OS [40]. Based on these experiences, we hypothesize that total tumor volume is a better determinant for prognostic assessment than the number of lesions in patients with BM. In our results, similar to the JLGK0901 study, we found that having a CITV value lower than 10 cc was associated with better OS.

GI tract tumors include esophageal cancer, gastric cancer, colorectal cancer, and other tumor types that differ in their biological behavior. Therefore, it appears logical to tailor the treatments or treatment options with respect to even more specific grading systems. In addition, as shown in our study, treatments such SRS which continue to advance with advancing technology should be more frequently used.

The limitations of this study include its retrospective design with the small sample size and heterogeneity of the patient population in terms of the origin of primaries. However, we showed that the SRS treatment was superior in patients with fewer BM lesions and lower intracranial metastasis volumes. Nevertheless, considering the current limitations, it would be erroneous to state definite conclusions, particularly because we did not assess variations in genetic characteristics and the details of systemic treatment in the current study.

### Conclusions

In patients with BMs resulting from primary GI cancers, SRS should be preferred in those with longer OS expectancy who have controlled extracranial disease, good KPS and CITV values of <10 cc. In patients with poor prognostic factors and short survival expectancy, WBRT can be applied without expecting the prevention of neurocognitive function decline. Furthermore, CITV appears to be an important determinant for patients with BMs from GI tumors treated with SRS or WBRT, and the total tumor volume should be examined more closely in future studies. The studies should continue to investigate prognostic factors, optimal dose fractionation schemes, treatment modalities, and side effects.
